# Persistent Organic Pollutants in Tagus Estuary Salt Marshes: Patterns of Contamination and Plant Uptake

**DOI:** 10.3390/jox14030066

**Published:** 2024-09-02

**Authors:** Ricardo Cruz de Carvalho, João Cardoso, João Albuquerque Carreiras, Paula Santos, Carla Palma, Bernardo Duarte

**Affiliations:** 1 MARE—Marine and Environmental Sciences Centre/ARNET—Aquatic Research Network, Faculdade de Ciências, Universidade de Lisboa, Campo Grande, 1749-016 Lisboa, Portugal; jpmega55@gmail.com (J.C.); jgcarreiras@ciencias.ulisboa.pt (J.A.C.); baduarte@ciencias.ulisboa.pt (B.D.); 2 cE3c—Centre for Ecology, Evolution and Environmental Changes, Faculdade de Ciências da Universidade de Lisboa, Campo Grande, 1749-016 Lisboa, Portugal; 3 Instituto Hidrográfico, Rua das Trinas 49, 1249-093 Lisboa, Portugal; paula.santos@hidrografico.pt (P.S.); carla.palma@hidrografico.pt (C.P.); 4 BioISI—Biosystems and Integrative Sciences Institute, Departamento de Biologia Vegetal, Faculdade de Ciências da Universidade de Lisboa, Campo Grande, 1749-016 Lisboa, Portugal; 5 Departamento de Biologia Vegetal, Faculdade de Ciências da Universidade de Lisboa, Campo Grande, 1749-016 Lisboa, Portugal

**Keywords:** pesticides, bioaccumulation, contamination, ecotoxicology, biomarkers

## Abstract

The presence of anthropogenic compounds, including organochlorine pesticides (OCPs) and polychlorinated biphenyls (PCBs), was studied in three salt marshes within the Tagus estuary, Portugal, along an anthropogenic pressure gradient. Results revealed differences in OCPs and PCBs among the marshes, with differing concentration levels. Specifically, one marsh, with surrounding agricultural activity, showed the highest OCP concentrations, while another, with a historical industrial past, exhibited elevated PCB levels. In contrast, a third marsh, part of a natural reserve, displayed comparatively lower concentrations of both substances. Sediment concentrations, likely influenced by agricultural practices, were found to be comparable to or higher than those observed in other Portuguese estuaries. The halophyte Spartina maritima was found to absorb OCPs, particularly in its aboveground tissues, suggesting bioaccumulation within the plant. Additionally, PCB levels appeared to be influenced by industrial history, with one marsh displaying notably higher concentrations. In conclusion, the persistence of organochlorine compounds in the salt marsh ecosystems notwithstanding the regulatory prohibitions implemented in the 1990s highlights the need for continuous monitoring and study of such sites and the necessity of remediation practices, which are imperative to mitigate ecological and health risks in these polluted salt marshes.

## 1. Introduction

The hydrosphere encompasses about 70% of the Earth’s surface, manifesting in diverse forms, such as seas, oceans, rivers, and lakes [1]. These aqueous domains play an essential function in sustaining terrestrial life, affording myriad ecological advantages. Notably, they contribute appreciably to international oxygen production, surpassing 50%, facilitated by the metabolic activities of marine flora, encompassing plant life, algae, and phytoplankton [2]. Furthermore, these aquatic habitats function as habitats, shelters, and feeding grounds for a myriad of fauna [3,4]. Of specific ecological significance are estuaries, vital biomes located at the confluence of freshwater and saltwater, forming brackish water and receiving nutrients from river basins that foster heightened productivity and biodiversity [5,6,7]. Estuarine ecosystems play a pivotal role in assisting diverse aquatic life forms, including fish, molluscs, crustaceans, and migratory birds [8]. They also provide valuable services for human activities, such as tourism and fishing, as well as environmental education opportunities as natural laboratories [9]. The flora of these biomes includes various species of macroalgae, seaweed, plants, and phytoplankton [10,11]. However, these ecosystems are highly vulnerable to anthropogenic impacts [12,13], such as coastal erosion, habitat loss and fragmentation, introduction of invasive species, disturbance to wildlife, and industrial pollution. In Portugal, these ecosystems are particularly at risk as they are located in densely populated coastal areas [14].

Salt marsh ecosystems, located in estuarine and coastal regions, host vegetation species that exhibit tolerance to salinity and submerged conditions [15,16,17]. These halophytic species, characterized by their ability to endure salt concentrations surpassing 200 mM, display a remarkable resilience to diverse environmental stressors, such as flooding and pollutants [18,19,20,21]. Functionally, salt marshes act as natural buffers against floods and coastal erosion, attenuating wave power and minimizing the threat of storm surges [18]. The halophytic vegetation in these marshes supports sediment deposition during high tide, primarily composed of fine-grained particles with a noted affinity for contaminants [22,23,24]. Notably, salt marsh plant life has advanced adaptive mechanisms to contend with pollution, serving as a natural remediator by sequestering potentially toxic compounds in sediments and biomass [25,26,27]. These ecosystems encompass a diverse array of halophytic species, including *Halimione portulacoides*, *Salicornia* spp., *Sarcocornia* spp., and *Spartina maritima*, prevalent in the Mediterranean and Atlantic systems [28]. The global distribution and the pioneering role of the Spartina genus, known as cordgrasses, underscores its success as a halophyte. This adaptability is reinforced by the high physiological and ecological plasticity of this genus, which renders it a potential biomonitor and exhibits a high phytoremediation potential [29,30,31,32].

However, despite the vital ecological functions of these ecosystems and the global distribution of halophytic species, there remains a concerning knowledge gap regarding the accumulation dynamics of anthropogenic compounds, such as organochlorine pesticides (OCPs) and polychlorinated biphenyls (PCBs), in salt marshes and their resident flora [33]. Characterized by high lipophilicity, low water solubility, and high solubility in fats, oils, lipids, and non-polar solvents [34,35,36,37], OCPs and PCBs are widely recognized as persistent pollutants [38]. The multitude of sources of these compounds, encompassing discharges, effluents, landfills, incineration, and combustion [39,40],contribute to their ubiquity in worldwide natural sinks, particularly in estuarine matrixes. The propensity of OCPs and PCBs to bioaccumulate in living organisms, thereby influencing the complete trophic chain, accentuates their ecological importance [41]. Despite their presence, comprehensive knowledge of the accumulation dynamics of these compounds in salt marshes remains elusive, necessitating dedicated research efforts [42]. Even though this is a relevant issue, there are few records in the literature on the presence of these pollutants in salt marsh ecosystems and vegetation. Some early studies have revealed that lighter PCBs can accumulate and translocate in *Spartina alterniflora* [43,44,45] as well as the macrophyte *Cladophora glomerata*, accumulating PCBs at levels similar to water [46]. Over the last ten years, additional research has determined that *Sarcocornia perennis* and *Halimione portucaloides* roots accumulate PCBs, but their upward movement is restricted [47]. More recent investigations have noted that species such as *Sarcocornia fruticosa*, *H. portucaloides*, and *Spartina maritima* are also capable of uptaking OCPs [48]. These initial results help us understand that contaminants can build up in salt marsh ecosystems; however, further research is needed to completely grasp the scope of the buildup, as well as its dynamics in and effects on these environments.

In light of the current knowledge gap, this study is focused on the halophyte *S. maritima* in Tagus estuary (Portugal), a site characterized by different anthropogenic pressures. The study examines whether PCBs and OCPs are present in salt marsh sediments and *S. maritima* to obtain basic knowledge of pollution levels while simultaneously showing how plants interact with their environment through sediment. Furthermore, this study aims to provide substantial additional information about the levels of persistent pollutants in certain salt marsh ecosystems over a human activity gradient, looking at the plant’s contribution to the uptake of contaminants from sediments, which will aid in understanding what the starting exposure values for primary consumers at the bottom of the food chain are like. These ideas are important for decision-making and in the formulation of effective conservation policies towards safeguarding the uniqueness of biodiversity and general health within such coastal habitats. Consequently, these results are crucial for managing and conserving them as advised by policymakers or other concerned authorities.

## 2. Materials and Methods

### 2.1. Plant Material and Collection Sites

The Tagus estuary is Portugal’s largest estuarine system and one of the largest wetlands in Europe. It is a highly diverse and productive ecosystem, harbouring various species of fish (e.g., sea bass, sole, lamprey, and eel), molluscs (e.g., clam and oyster), crustaceans (e.g., shrimp, prawn, crab), and many birds (e.g., pied avocet, flamingo, greylag goose, dunlin, heron, stork), as well as macroalgae (e.g., *Ulva lactuca*, *Gracilaria gracilis*, *Cladophora prolifera*), plants (e.g., *Sarcocornia perennis*, *S. fruticosa*, *Halimione portulacoides*, and the endemic *S. maritima*), and phytoplankton (e.g., diatoms, cryptophytes, and dinoflagellates) [49,50,51,52,53]. In the south margin, it spreads out into three sections: the Tagus estuary natural reserve, which encompasses the Benavente and Alcochete municipalities; the Moita and Sarilhos bays, which include the Moita, Alhos Vedros, Gaio-Rosário, and other locations; and the Seixal and Coina bays, which cover Corroios, Amora, Seixal, and Coina [54].

**Figure 1:**
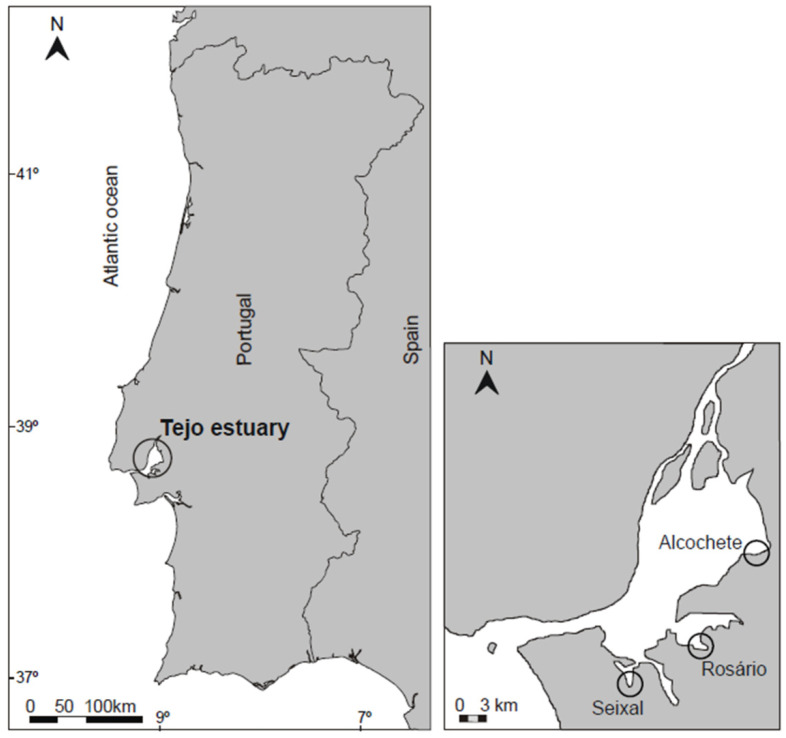
Tagus estuary map with the detailed location of the three sampling sites.

Sediments and plant samples were collected during low tide in November 2020 at three salt marshes located in the Tagus estuary with different degrees of anthropogenic pressure [55,56]: Alcochete (38°45.661′ N, 8°56.116′ W, Natural Reserve, population density of 149.1/km^2^), Rosário (38°40.161′ N, 9°0.198′ W, population density of 1199.0/km^2^), and Seixal (38°38.313′ N, 9°07.191′ W, population density of 1744.4/km^2^) (Figure 1). *Spartina maritima* plants and sediments were sampled (*n* = 5 per site). The sediment samples (0–5 cm depth) were collected around the plants (rhizosediments) with a stainless-steel scoop and deposited into a plastic sample container. Samples were brought back to the laboratory in refrigerated bags and stored at −20 °C until further analysis.

### 2.2. Sample Pre-Processing

Plant samples were washed with milli-Q water to remove sediment particles, algae, or other unwanted particles, divided into aboveground and belowground parts, and sliced in a Magic Bullet blender (Capbran Holdings LLC, Los Angeles, CA, USA).

Rhizosediment samples were divided into two fractions: one for granulometry analysis, and the other for the analysis of total organic carbon (TOC), humidity, and organic matter, organochlorine pesticide (OCP), and polychlorinated biphenyl (PCB) concentrations. The latter fraction was fractionated with a sieve with a mesh of 2 mm, and samples were frozen at −20 °C in Petri plates. Afterwards, samples were freeze-dried at approximately 5 μm Hg of pressure and −40 °C (LyphLock 1 L, Labconco, Kansas City, MO, USA) for up to four days. Thereafter, samples were dried, followed by milling with mortar grinders RM 200 (Retsch, Haan, Germany) and PULVERISETTE 2 (Fritsch, Idar-Oberstein, Germany).

### 2.3. Granulometry Analysis

The granulometry analysis was performed through two methods: sifting [57,58,59], and the laser diffraction technique [60]. To remove organic matter, sediment samples were submitted to 30 and 60-volume solutions of hydrogen peroxide (H_2_O_2_). Subsequently, the samples were heated in a water bath at 80 °C in a flux chamber with gas extraction for between five and nine days to ensure full organic matter removal and evaporation of excess H_2_O_2_. During this process, samples were manually shaken several times a day, ensuring that the samples stayed hydrated by adding distilled water. Afterwards, the samples were cleaned of the dissolved salts by vacuum using porcelain containers, repeating this procedure five times. Subsequently, the samples were fractionated in a sieve column (31.5 to 0.063 mm of mesh). For the fractionation of samples with granulometry inferior to 0.5 mm, the laser diffraction technique was used, resorting to the MALVERN Mastersizer Hydro 2000 G (Malvern Panalytical Ltd., Malvern, UK).

### 2.4. Total Organic Carbon

The total organic carbon (TOC) of the samples was determined through the difference between the total carbon and the total inorganic carbon. Total carbon was determined by converting it into carbon dioxide (CO_2_) through combustion, while total inorganic carbon was determined through acidification of the sample followed by purge of the CO_2_ [61]. Both these procedures were performed and then quantified in an elemental analyser of carbon and nitrogen, SKALAR Primacs SN100907 (Skalar Analytical B.V., Breda, The Netherlands). Absorbance measurements were made by non-dispersive infrared absorption spectrometry.

### 2.5. OCPs and PCBs Extraction and Quantification

The OCPs (Aldrin, DDE [dichlorodiphenyldichloroethylene], DDT [dichlorodiphenyltrichloroethane], HCB [hexachlorobenzene], Heptachlor-epoxide, and trans-Nonachlor) and PCBs (PCBs 28, 52, 101, 105, 118, 138, 153, 156, and 180) were extracted from the sediments through the accelerated solvent extraction technique (ASE) using a Dionex Accelerated Solvent Extractor (Thermo Fisher Scientific Inc., Waltham, MA, USA). Approximately 15 g of sediments was mixed with approximately 8–9 g of diatomaceous earth (DE) to fill the cell [62]. The extraction operating conditions are presented in Table S1 (Supplemental Information). At the end of the process, activated copper was added to the samples to remove sulphur and, thus, remove interference in the analysis of OCPs and PCBs [63]. The samples were concentrated in a Heidolph Laborota 4000 rotary evaporator (Heidolph Instruments GmbH & Co. KG, Schwabach, Germany), avoiding complete dryness, which could cause partial or total loss of the analytes. Afterwards, the samples were placed under nitrogen flow until they were concentrated at approximately 2 mL. Next, the samples were purified using a column of silica gel and basic alumina, both deactivated at 5% using milli-Q water. This column was made of glass wool, 5 g of silica gel, 5 g of basic alumina, and approximately 1 cm thick anhydrous sodium sulphate, using n-hexane as a solvent. The packing of the column was constructed carefully to prevent the formation of fissures and air bubbles in it, and it was also ensured that the column was never dried. After the addition of the sample, the column was cleaned with two doses of 5 mL each of n-hexane, and after the second extractant, 25 mL of n-hexane was added. After this step, the samples were concentrated in the rotatory evaporator as previously described. Then, the solution was transferred to 10 mL tubes, and the necessary amount of isooctane was added to bring the volume to triple the concentrated extract. Afterwards, the samples were dried with nitrogen flow until reaching 1 mL of volume. The extraction of OCPs and PCBs contained in the below- and aboveground parts of the *S. maritima* previously sampled by the QuEChERS methodology was used [64]. Firstly, approximately 15 g of plant sample was transferred to a 50 mL tube, and 15 mL of 1% acetic acid in acetonitrile was added. Subsequently, 4 g of anhydrous magnesium sulphate, 1 g of sodium chloride, 1 g of monosodium citrate, and 0.5 g of sodium citrate dibasic sesquihydrate were added, and the tubes were shaken in the vortex for 1 min and centrifuged at 1500 rpm for 1 min. Afterwards, 5 mL of supernatant was transferred to another 50 mL tube and a clean-up salt mixture was added. We used 150 mg of MgSO_4_ to absorb the remaining water in the extract, 25 mg of primary-secondary amine (PSA) to eliminate the high concentrations of sugars, organic acids, or fatty acids contained in the sample, and 2.5 mg of graphitized carbon black (GCB) to remove the elevated levels of pigments. The tube was shaken at the vortex for 30 s and centrifuged at 1500 rpm for 1 min [64]. Afterwards, the sample was concentrated with a nitrogen flow to almost complete dryness, and, finally, 1 mL of isooctane was added to the sample. Before being put in vials, 25 μL and 100 μL of internal standard (PCB155 and PCB198) were added to the plant extract and to the sediments extract, respectively, to facilitate the analysis of the peaks by gas chromatography with an electron capture detector (GC-ECD), according to ISO 10382 [65]. In this procedure, five standard solutions were also prepared, each with a different known concentration (2 ng mL^−1^–12 ng mL^−1^ for PCBs and 0.8 ng L^−1^–4.80 ng^−1^ for OCPs), for the calibration of the method. Sample quantification was performed through GC-ECD (Hewlett Packard 6890 Series GC gas chromatograph equipped with ^63^Ni-ECD for sediments, and Agilent 8890 GC System for plants extracts) using a fused silica CP-Sil 8 CB column (60 m length, diameter 0.25 mm diameter, 0.1 μm film thickness) [66]. The operating conditions are presented in Table S2 (Supplemental Information). The peaks corresponding to each OCP or PCB were identified according to the corresponding retention time (Table S3, Supplemental Information). To quantify each analyte, the following expression was used:

$$x=\frac{y}{b}\times \left(\frac{V_{ext}\times 1000}{V_{inj}}\right)\times \frac{1000}{m_{A}}\times \frac{100}{W_{dry}}\times \frac{100}{REC_{x}}$$

where *x* is the concentration of *x* analyte (μg Kg^−1^), *y* is the instrumental sign (peak’s height) expressed in Hz, *b* is the slope of the linear calibration used in this experimentation, *V_ext_* is the final volume of the organic extract (in this case, 1 mL), *V_inj_* is the volume injected into the column (in this case, 2 μL), *m_A_* is the initial mass of the sample, *W_dry_* is the dried weight of the sample, and *REC_x_* is the recovery of the analyte *x*. To ensure quality control of OCP and PCB analysis in sediments and plants, a blank sample of the procedure was made, and a fortified blank sample and duplicate samples were prepared. For blank and fortified samples, only DE was added in the extraction cell, and in the case of the fortified blank, 100 μL of the fortified standard was added. Fortified samples were also made to evaluate the quality of the experiment. Using this procedure, the recovery of the test could be calculated using the following expression:

$$REC_{x}\left(\%\right)=\frac{x_{BF}}{x_{t}}\times 100$$

where *REC_x_* is the recovery of *x* analyte in the organic extract in an inert matrix, *x_BF_* is the concentration of *x* analyte in the fortified blank (in the case of the plant sample, it is the concentration of the fortified sample minus the concentration of the non-fortified sample), and *x_t_* is the theoretical concentration of *x* analyte that was initially on the fortified solution added to the inert matrix. The criterion for acceptance of the blank samples was that their concentration should be inferior to the limit of detection (LD) of the method. In the case of the recovery tests, the criterion was that its value should be between 70% and 130%. For the duplicate tests, the acceptance criterion considered was that the relative difference between the results should be inferior to 19.5%.

Furthermore, sediment matrix contamination was assessed by comparing the obtained values to sediment quality guidelines (SQGs) established by regulatory bodies such as the United States Environmental Protection Agency (US EPA) and the Convention for the Protection of the Marine Environment of the North-East Atlantic (OSPAR) [67,68] (Table 1).

### 2.6. Statistical Analysis

Since the data lacked normality and homogeneity of variances, the statistical analysis was based on a non-parametric Kruskal–Wallis analysis of variance of each of the attained variables. Bonferroni post hoc tests were performed using the ‘agricolae’ package to compare the same variable among samples with different origins, considering statistical significance at *p* < 0.05. Boxplots were employed using the ‘ggplot-2’ package in R-Studio Version 4.1.2 [69]. Principal component analysis was performed using the ‘ggfortify’ package [70], and Spearman correlations were determined using the ‘corrplot’ package [71].

## 3. Results

### 3.1. Sediment Composition

**Figure 2:**
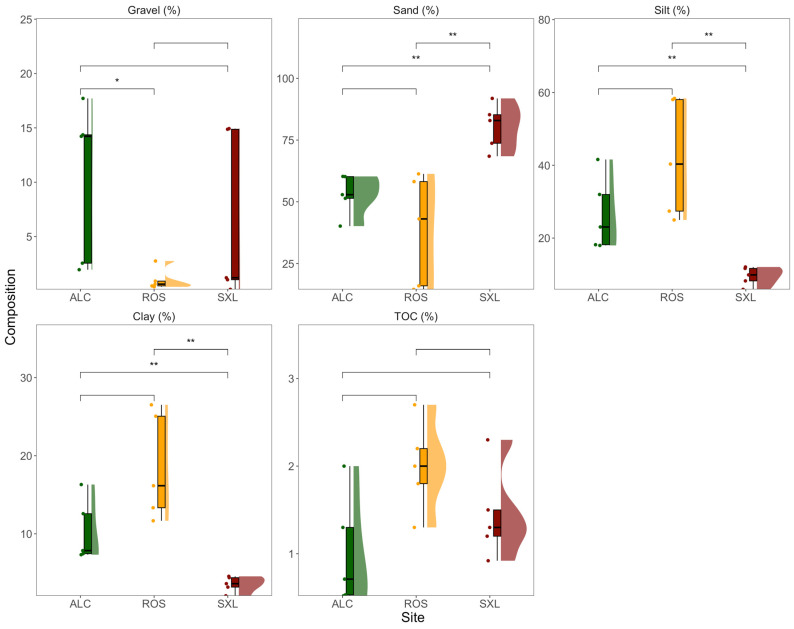
Gravel, sand, silt, clay, and total organic carbon (TOC) relative composition in the sediment samples from the three sampling sites (ALC, Alcochete; ROS, Rosário; SXL, Seixal) in the Tagus estuary (*n* = 5, boxplots with average ± standard deviation, asterisks denote statistical differences at *p* < 0.05 * and *p* < 0.01 **).

Disparities in sediment particle composition were discerned among the triad of locations (Figure 2). Alcochete and Rosário exhibited a notably analogous composition, with the sole distinction of lower gravel content at the Rosário site. In contrast, Seixal displayed a distinct composition characterized by a significantly higher proportion of sand and significantly lower percentages of silt and clay compared to the other two sites. Statistical analyses revealed no significant disparities in total organic carbon content across the three sites.

### 3.2. OCPs and PCBs in Sediments and Plants

**Figure 3:**
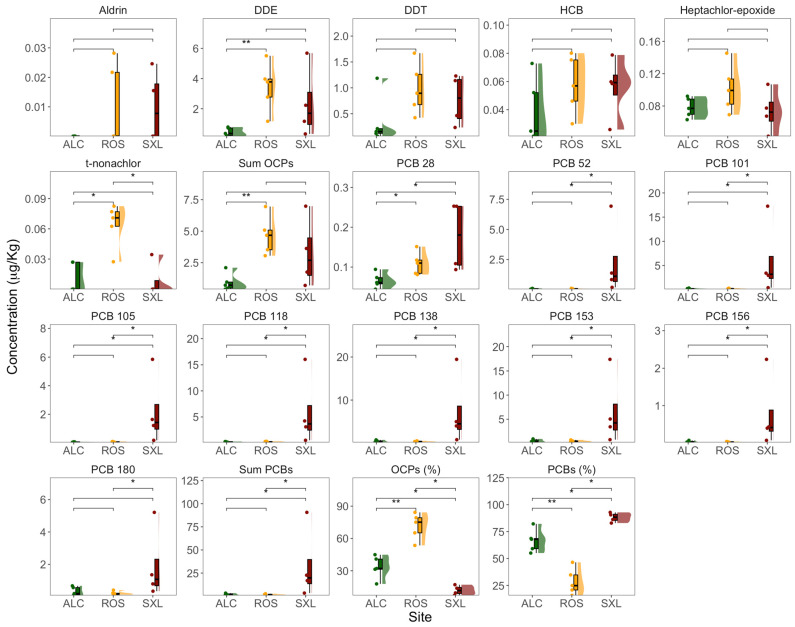
Concentration (µg/Kg) and distribution of OCPs (aldrin, DDE [dichlorodiphenyldichloroethylene], DDT [dichlorodiphenyltrichloroethane], HCB [hexachlorobenzene], heptachlor-epoxide, and trans-Nonachlor), and PCBs (PCB 28, 52, 101, 105, 118, 138, 153, 156, and 180) in the sediment samples from the three sampling sites (ALC, Alcochete; ROS, Rosário; SXL, Seixal) in the Tagus estuary (*n* = 5, boxplots with average ± standard deviation, asterisks denote statistical differences at *p* < 0.05 * and *p* < 0.01 **).

The examination of OCP and PCB concentrations in the collected sediment samples unveiled discernible distinctions among the scrutinized sampling sites (Figure 3). Regarding OCPs presence, the analysis indicated that aldrin, DDT, heptachlor-epoxide, and HCB exhibited no statistically significant differences in sediment concentrations across all three sampling sites. In contrast, DDE concentrations were found to be higher in Rosário sediments relative to Alcochete. Trans-Nonachlor displayed higher concentrations in the sediments collected at Rosário marsh when juxtaposed with the other two sites. Seven PCB congeners (PCB 52, 101, 105, 118, 138, 153, and 156) were found to be significantly higher in the sediments of Seixal compared to the other two marshes. On the other hand, the concentration of PCB 180 was significantly lower in Rosário when compared to Seixal. Additionally, the sum of PCB concentrations was highest at the Seixal site. In terms of relative concentrations, Alcochete displayed a distribution of approximately 40% OCPs and 60% PCBs, while Rosário exhibited a ratio of about 75% OCPs to 25% PCBs. Notably, in Seixal, PCBs dominated the measured pollutants, constituting around 85% of the total.

**Figure 4:**
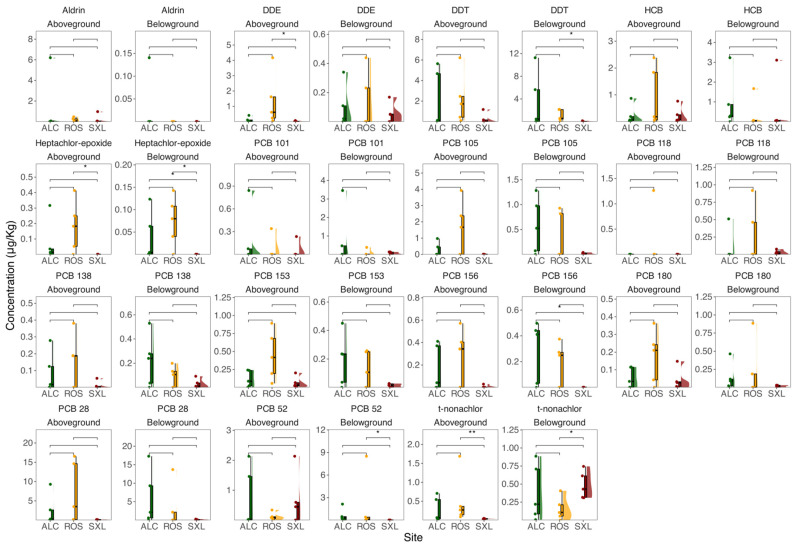
Concentration (µg/Kg) and distribution of OCPs (aldrin, DDE [dichlorodiphenyldichloroethylene], DDT [dichlorodiphenyltrichloroethane], HCB [hexachlorobenzene], heptachlor-epoxide, and trans-Nonachlor) and PCBs (PCB 28, 52, 101, 105, 118, 138, 153, 156, and 180) in plant aboveground and belowground organ samples from the three sampling sites (ALC, Alcochete; ROS, Rosário; SXL, Seixal) in the Tagus estuary (*n* = 5, boxplots with average ± standard deviation, asterisks denote statistical differences at *p* < 0.05 * and *p* < 0.01 **).

Examining the concentrations of OCPs and PCBs in plant tissues revealed certain distinctions (Figure 4). Regarding OCPs presence, the analysis demonstrated that aldrin and HCB exhibited no statistically significant differences across all three sampling sites in the above- and belowground plant organs. Conversely, DDT displayed elevated concentrations belowground in Rosário relative to Seixal, while DDE showcased higher values aboveground in Rosário compared to Seixal. The concentration of heptachlor-epoxide in the belowground environment was significantly lower in Seixal compared to the other two sites. However, aboveground, the concentration was only significantly lower in Seixal when compared to Rosário. On the other hand, trans-Nonachlor displayed increased concentrations aboveground, but decreased concentrations belowground in Rosário when compared to Seixal. Remarkably, the analysed PCBs exhibited no significant variation in the sampled plant tissues across all three sampling sites. However, there were two exceptions, with PCB 52 showing significantly higher concentrations belowground in Rosário compared to Seixal, and PCB 156 demonstrating significantly higher concentrations belowground in Alcochete when compared to Seixal.

**Figure 5:**
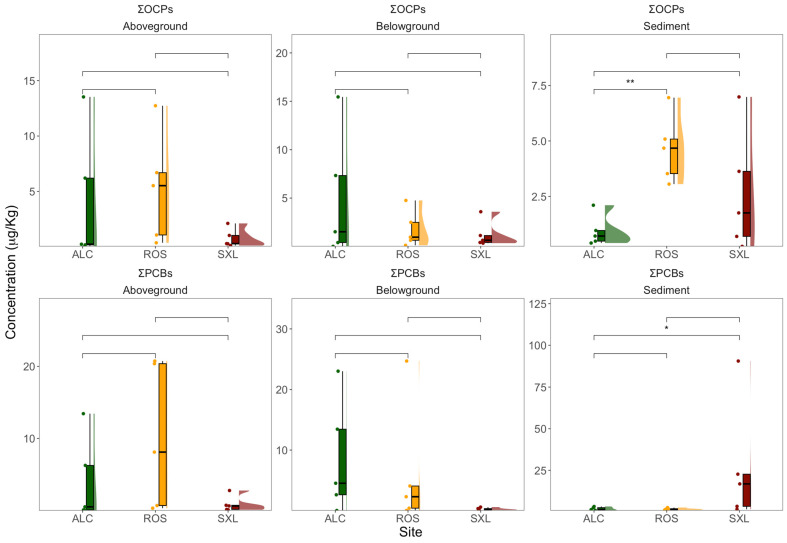
Sediment and plant (above- and belowground) OCP (ΣOCPs) and PCB (ΣPCBs) overall concentration (µg Kg^−1^) in samples collected at three sampling sites (ALC, Alcochete; ROS, Rosário; SXL, Seixal) in the Tagus estuary (*n* = 5, boxplots with average ± standard deviation, asterisks denote statistical differences at *p* < 0.05 * and *p* < 0.01 **).

The examination of distinct compound groups, encompassing ΣOCPs (the cumulative concentration of all OCPs) and ΣPCBs (the cumulative concentration of PCB 28, 52, 101, 105, 118, 138, 153, 156, and 180), elucidated noteworthy outcomes (Figure 5). Sediments procured from Rosário exhibited a significantly elevated value of ΣOCPs in comparison to Alcochete samples. Concerning classes, no statistically significant distinctions surfaced in the concentration of ΣOCPs. Regarding ΣPCB concentrations, significant differences were found between the sediment samples collected at Alcochete and Seixal, with the latter being higher.

### 3.3. Correlation and Multivariate Analysis

**Figure 6:**
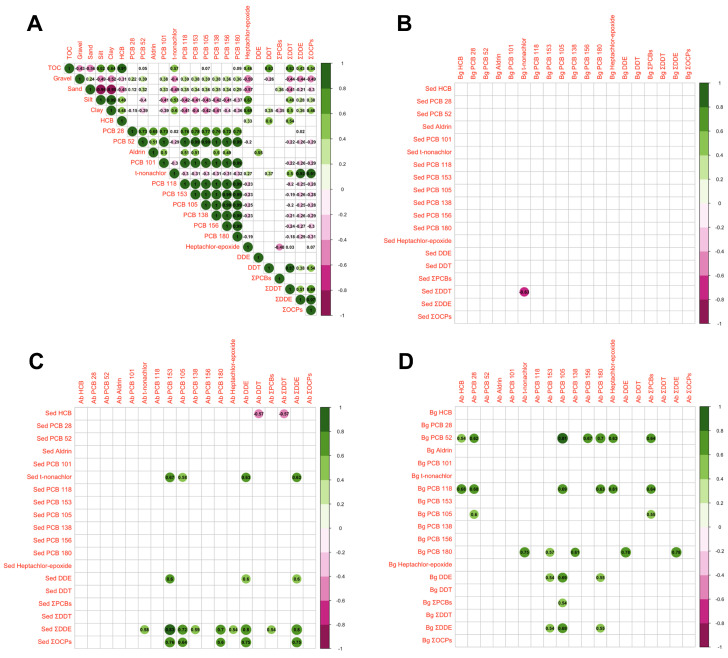
Spearman correlation coefficients between the sediment particle composition, OCPs, and PCBs (**A**), between OCPs/PCBs in sediments and the belowground plant organs (**B**), between OCPs/PCBs in sediments and aboveground plant organs (**C**), and between belowground and aboveground plant parts (**D**) of the three sampling sites (Alcochete, Rosário, Seixal) in the Tagus estuary (significant Spearman correlation coefficients values inside; only statistically significant correlations are shown).

The sediment particle composition and PCBs exhibited a positive correlation with gravel and sand while displaying a negative correlation with silt and clay (Figure 6A). In contrast, OCPs showed an opposite trend, leading to distinctive distributions of PCBs and OCPs along the sediment profile. A significant negative correlation was observed between ƩDDT in sediments and trans-Nonachlor in belowground plant parts when contrasting the sediments and belowground plant components (Figure 6B). Conversely, positive correlations were discerned between pollutants in sediments and those in aboveground plant parts, particularly ƩDDE with several PCBs (105, 138, 153, 180), encompassing ƩPCBs as well as trans-Nonachlor, heptachlor-epoxide, and ƩDDE (Figure 6C). Furthermore, a negative correlation was evident between HCB in sediments and DDT in belowground plant parts. Regarding the correlation between belowground and aboveground plant parts, the most noteworthy aspect was the positive correlation of belowground PCB52 and PCB118 with various aboveground PCBs, including ƩPCBs (Figure 6D).

**Figure 7:**
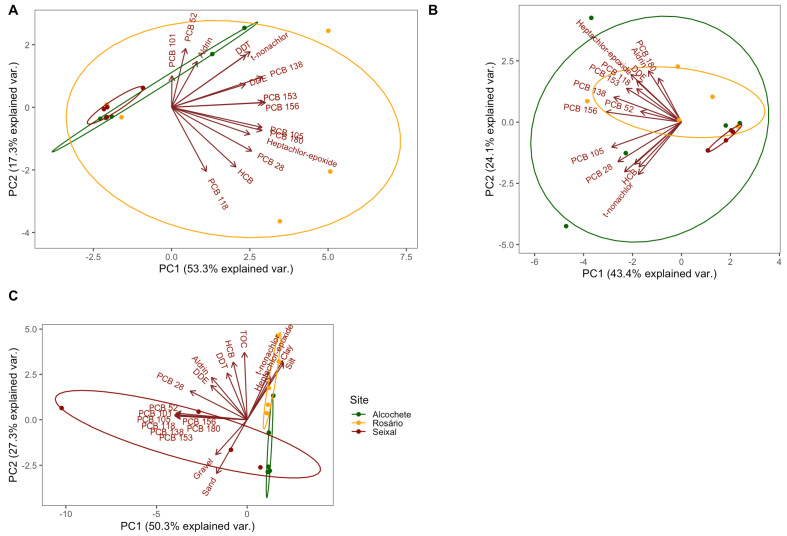
Principal component analysis (PCA) of OCPs (aldrin, DDE [dichlorodiphenyldichloroethylene], DDT [dichlorodiphenyltrichloroethane], HCB [hexachlorobenzene], heptachlor-epoxide, and trans-Nonachlor) and PCBs (PCB 28, 52, 101, 105, 118, 138, 153, 156, and 180) concentrations in aboveground (**A**) and belowground (**B**) plant organs and in sediments ((**C**), including granulometry and TOC [total organic carbon]) collected at the three sampling sites (Alcochete, Rosário, Seixal) of the Tagus estuary.

Examining the groups delineated through principal component analysis (PCA) of OCP and PCB concentrations in aboveground organs of *S. maritima* (Figure 7A), the cluster formed by the site with intermediate anthropogenic pressure (Rosário) displays substantial dispersion among its samples, overlapping the two groups represented by samples collected at Alcochete and Seixal salt marshes. A similar effect is observed when scrutinizing the PCA results for OCP and PCB concentrations in belowground organs of *S. maritima* (Figure 7B); however, in this instance, heightened dispersion is evident for samples collected at the salt marsh with a lower degree of anthropogenic pressure. Notwithstanding, it is noteworthy that, in both scenarios, samples collected at Seixal, the salt marsh with higher anthropogenic pressure, exhibit lower dispersion in the PCA biplot, signifying a greater degree of similarity between samples concerning PCB and OCP concentration.

The cluster distribution concerning sediment OCPs, PCBs, and characteristics (Figure 7C) elucidates a distinct separation along the first principal component axis (PC1), contributing to half of the explained variation (50.3%), and the second principal component axis (PC2), explaining approximately a quarter of the variation (27.3%). PCBs (excluding PCB28) and OCPs are markedly segregated, with PCBs associated with the gravel and sand component of the sediments, while other pollutants are more prevalent in the silt and clay components, validating the previously delineated distinctions.

**Figure 8:**
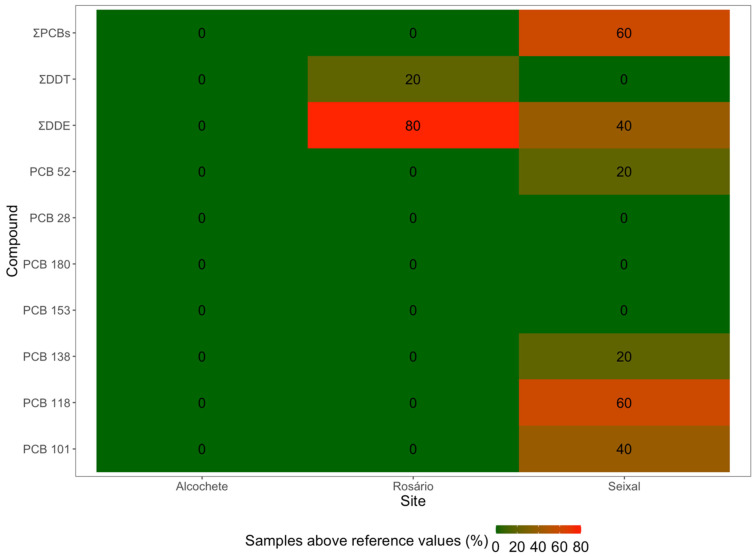
Percentage of samples above the reference values in the sediments at the three sampling sites (Alcochete, Rosário, Seixal) of the Tagus estuary (*n* = 5) for each of the compounds with established thresholds.

The concentrations of OCPs and ƩPCBs in the sediments of the surveyed salt marshes in the Tagus estuary were compared with the SQG values established by the United States Environmental Protection Agency (US EPA), and the individual PCBs were compared with the SQG values established by the Convention for the Protection of the Marine Environment of the North-East Atlantic (OSPAR), enabling an assessment of the contamination levels at each site (Figure 8). The findings indicated that most PCBs in Seixal sediments exceeded OSPAR SQGs in at least one sample (PCB 52, 101, 118, 138), also reflected in the exceeded values for ΣPCBs in the US EPA, with the presence of ΣDDE also noted. Rosário sediments exceeded the US EPA SQG for DDE in almost all samples (4 out of 5), and ΣDDT was found to be above the reference level in one sample. In contrast, Alcochete sediments exhibited lower susceptibility to these contaminants.

## 4. Discussion

Comparisons of DDE and DDT concentrations in the sediments of the three sites in Tagus salt marshes were conducted against values reported in previous studies for other Portuguese estuaries [72,73,74]. Gil and Vale [72] focused on the lower Tagus estuary and upper Sado estuary, while Carvalho et al. [73] investigated seven estuaries (Minho, Lima, Cávado, Ave, Douro, Sado, and Ria Formosa), and Lobo et al. [74] studied the Sado estuary. In our study, DDE concentrations varied between 0.18 and 0.79 μg kg^−1^ (Alcocehete), 1.17 and 5.51 μg kg^−1^ (Rosário), and 0.13 and 5.69 μg kg^−1^ (Seixal), while DDT varied between 0.07 and 1.19 μg kg^−1^ (Alchochete), 0.42 and 1.67 μg kg^−1^ (Rosário), and 0.12 and 1.23 μg kg^−1^ (Seixal). The DDE concentration ranged from 0.02 to 10.5 μg kg^−1^ in the Tagus estuary and from 0.02 to 2.13 μg kg^−1^ in the Sado estuary, and the DDT concentration ranged from 0.07 to 10.1 μg kg^−1^ and from 0.07 to 0.99 μg kg^−1^ in those locations [72]. In the other seven locations, the DDE concentration varied from 0.29 to 2.6 μg kg^−1^, while the DDT concentration varied from 0.27 to 3.9 μg kg^−1^ [73]. Values in the Sado for DDE ranged between 0.05 and 0.7 μg kg^−1^, and for DDT ranged between 0.7 and 4.4 μg kg^−1^ [74]. The results indicate that Rosário and Seixal exhibit higher DDE and DDT concentrations than the Sado estuary, while Alcochete demonstrates comparable concentrations [72,74]. Furthermore, the DDE and DDT concentrations in all three salt marshes fall within or below previously reported values for the Tagus estuary. Rosário and Seixal also had higher DDE concentrations than the seven estuaries studied in [73], while Alcochete had comparable concentrations.

Comparing our findings on a global scale highlights the variability of DDT and DDE concentrations across different estuarine systems. For instance, the An Hoa Estuary, is located in the Nui Thanh District, Quang Nam Province (Vietnam), which has a population density of 265.6/km^2^ (2019) [75] and contains several economic sectors such as agriculture, aquaculture, seaports, and waterway transportation. Upon examination, the estuary was shown to present DDE values which varied between 0.33 and 1.82 μg kg^−1^, and the DDT concentrations varied between 0.29 and 1.64 μg kg^−1^. In the latter case, the anticorrosive coatings of fishing boats could have been an additional source of DDT contamination [76], something that can also occur in the analysed sites of our study. In a study conducted on the Nile Delta (Egypt), population density in the analysed sites varied greatly between 588.7/km^2^ (Port Said) and 2412.0/km^2^ (Alexandria) (2023) [75], with economic activities which included agriculture, industry, and fisheries, and DDE concentrations ranging between 0.27 and 1.16 μg kg^−1^, while DDT varied between 0.004 and 3.83 μg kg^−1^ [77]. On the other hand, the Hangzhou Bay, Zhejiang Province (China) is a typical trumpet-shaped tidal estuary formed by the flowing of the Qiantang River into the East China Sea with several large cities such as Shanghai (population density of 4011/km^2^ (2020)), Jiaxing (population density of 1329/km^2^ (2020)), and Ningbo (population density of 1029/km^2^ (2020)) [75], and international ports, presenting commercial, agricultural, and industrial activities and heavy cargo transportation. At this estuary, DDE ranged from 0.47 to 1.04 μg kg^−1^ and DDT varied between 7.92 and 17.21 μg kg^−1^ [78]. Nevertheless, these concentrations are much lower than those found in the Kosi Bay (South Africa), an area where the major economic activities are eco-tourism, fishing, and agriculture and where there is low population density (between 37.97 and 109.6/km^2^ (2022)) [75], but where DDE can vary between 14.2 and 122.8 μg kg^−1^ and DDT varies between 4.5 and 51.8 μg kg^−1^ [79]. Thus, bearing in mind this information from various estuaries worldwide, including the Tagus estuary, it is evident that local sources of pollution, regional environmental conditions, and socioeconomic factors strongly influence the occurrence and distribution of these contaminants. There is, thus, a need for continuous monitoring and international cooperation to enhance the current understanding and management of risks associated with these persistent pollutants in different estuarine ecosystems.

The sediments from Alcochete salt marsh, characterized by a higher proportion of larger grains, as indicated by their gravel percentage, may be attributed to the marsh’s lower maturity degree, larger grains, and lower organic matter content [80]. Additionally, its upstream location tends to favour the deposition of heavier sediments, i.e., larger grains, as the river loses energy from upstream to downstream [81]. Conversely, the Rosário site exhibits higher OCP concentrations due to its proximity to agricultural activities and finer sediments (clay and silts). The inverse relationship between OCP concentrations and sediment particle size indicates higher sorption capacity in finer particles, influenced by the structural hysteresis of clay fractions, hindering the diffusion of pesticides from micropores [82,83,84]. Despite this, *Spartina maritima* mobilizes a portion of the OCPs to its tissues, particularly aboveground, with heightened concentrations observed in the Rosário salt marsh, where OCP contamination is more pronounced. These findings align with prior research [85,86,87], which reported varying OCP concentrations in plants grown in polluted soils. These OCPs can be transferred into plants through various processes, such as volatilization, soil particle movement, and plant transport, accumulating in the vegetation and storing them, which can be a way to remove them from the soils through phytoremediation [88], but can also resulting in their entry into food chains [89].

Comparative analysis of seven PCB concentrations in the sediments of the three salt marshes in the Tagus estuary against previous studies for the Tagus prodelta [90] and the Sado Estuary [74] shows distinctions. While in the Tagus prodelta sediments, reported PCB concentrations ranged from 0.3 to 30.6 μg kg^−1^ (with PCBs 138, 153, and 180 being the most dominant), in the Sado estuary sediments, reported PCB concentrations ranged between 0.08 and 3.4 μg kg^−1^ (with PCBs 138 and 153 being the most dominant). The results of this study showed that the PCB concentrations in the sediments of Alcochete and Rosário were lower than those observed for the Tagus prodelta and Sado estuary (ranging from 0.9 to 3.3 μg kg^−1^). On the other hand, in the sediments collected at Seixal salt marsh, the PCB concentrations were much higher, ranging from 1.6 up to 90.6 μg kg^−1^, much higher than in the Sado estuary and within range of or higher than those reported for Tagus prodelta, with the most dominant PCBs in Seixal being PCB 118, 138, and 153. These differences reflect the specific activities at each site: Alcochete, with minimal agricultural and industrial operations, contrasts with Rosário’s extensive agricultural practices, employing OCPs like DDTs, and Seixal’s industrial activities utilizing PCBs [91]. Our analysis revealed varying degrees of OCP and PCB contamination across the studied salt marshes within the Tagus estuary. Seixal sediments displayed the most concerning levels, exceeding established SQGs for several specific PCBs (52, 101, 118, 138) under the OSPAR framework, and ΣPCBs under the US EPA OSPAR framework. This suggests significant historical and potentially ongoing OCP and PCB input at this site, further supported by the presence of ΣDDE, a DDT breakdown product. Rosário sediments exhibited elevated DDE levels exceeding US EPA SQGs in most samples, indicating localized DDT contamination. However, ΣDDT remained within reference levels, suggesting limited recent input. In contrast, Alcochete sediments presented the lowest contamination levels, suggesting less historical and contemporary exposure to these persistent organic pollutants. This differential pattern across the estuary highlights the importance of site-specific assessments for understanding OCP and PCB contamination. Further investigations should be addressed to pinpoint the sources and transport pathways contributing to these differences, particularly at Seixal and Rosário. Additionally, exploring potential ecological and human health risks associated with the observed contamination levels is crucial for informing effective management strategies.

Evaluation of PCB and OCP accumulation trends in plants relative to sediment concentrations reveals a direct link between DDE sediment and aboveground concentrations, indicating a potential direct influence of sediment concentration on plant DDE bioaccumulation. The lack of correlation between other analysed OCPs and PCB concentrations in sediments and plants may result from metabolization of the parent compounds by plant metabolism. Research indicates that certain plant species possess the capacity to metabolize organochlorine pesticides and PCBs accumulated in their organs. Previous studies [92] have demonstrated the PCB biodegradative ability of plant cells, with *Solanum nigrum* showing the highest capability. Additionally, aquatic plants have been found to accumulate and metabolize organophosphorus pesticides [93], with several PCB congeners and metabolites identified in black nightshade hairy root culture [94]. This metabolization capacity was found to be species-specific, with different plant species having varying capacities for metabolizing PCBs [95]. While an avoidance mechanism cannot be excluded, studies collectively suggest that plants can metabolise these compounds, offering a potential natural solution for their removal from the environment. This aligns with *S. maritima*’s recognized characteristics as a phytostabilizer species, retaining OCPs and PCBs in its sediments due to sediment physicochemical alterations, akin to its behaviour with heavy metals in rhizospheric sediments [22,96,97]. Nevertheless, further investigations are warranted regarding congener distribution in plant tissues and potential sediment phytostabilization mechanisms. Other Spartina species, including *Spartina alterniflora* Loisel, have demonstrated the capacity to accumulate PCBs and OCPs, emphasizing the need for ongoing monitoring of these compounds in salt marsh vegetation [44]. This author reinforces the relevance of the presence of these pollutants in *Spartina* species as a potential source and vector for the mobilization of sediment-bound chlorinated hydrocarbons into the estuarine food chain, reinforcing the need for monitoring these compounds in salt marsh vegetation [44]. A survey conducted in a coastal area in Galicia (Spain) detected similar values of PCBs and OCPs as the ones detected here in coastal plant tissues, alongside several degradation products [98]. On the other hand, a previous study in Argentina detected much lower values than those verified in the surveyed Tagus estuary salt marsh plant samples, with Bulrush roots accumulating OCP concentrations of 30.2–45.7 µg kg^−1^ of plant dry weight [99]. The observed concentrations of PCBs and OCPs in plant samples appear to be species-dependent, underscoring the importance of surveying the most abundant estuarine halophytes to identify optimal biomonitoring species. However, the trends in OCP and PCB levels align with those observed for other contaminants in the Tagus estuary, reflecting higher concentrations in sediments where increased levels of other legacy contaminants have been reported.

The occurrence and distribution of contaminants within sediments and distinct plant components of halophytes are intricately linked to the physicochemical attributes of the sediments, thereby influencing the spatial dynamics of halophyte vegetation in salt marsh ecosystems [33,100]. Furthermore, these contaminants exhibit the potential for bioaccumulation in both the roots and photosynthetic organs of diverse plant species, presenting consequential ecological and health implications [27,44,101]. Essentially, the bioaccumulation and biomagnification of contaminants such as OCPs and PCBs are dangerous to the health of ecosystems and their aquatic food webs [102,103,104]. The facts on bioaccumulation and biomagnification of pollutants in various species of different living beings have been established in fish, birds, and mammals (see review in [105]). For instance, OCPs and PCBs can accumulate in the tissues of organisms, resisting biodegradation and leading to long-term exposure and potential toxic effects [106]. This is even more troublesome for large predators, since they tend to accumulate high amounts of toxins given their position in the food web [107,108,109]. Moreover, despite the effects on the fauna, these specific pollutants are dangerous for people who consume fish, posing threats to human health in the form of endocrine disruption, developmental problems, and increased rates of cancer [110]. Thus, the assessment and regulation of pollutant concentrations in salt marsh environments is important for preserving fauna and people from the adverse effects of pollutants.

In summary, OCPs and PCBs, classified as persistent organic pollutants, have been banned in Portugal since the late 1970s. However, these compounds continue to persist in the environment due to their prolonged half-lives and limited biodegradability. Portuguese salt marshes, recognized as pivotal coastal ecosystems providing diverse ecological services, face exposure to these contaminants through runoff, wastewater discharge, and atmospheric deposition [111]. The concentrations of OCPs and PCBs in salt marshes exhibit variability based on land use and anthropogenic activities in their proximate surroundings [112]. In particular, the sediments of the Rosário salt marsh, located in an area characterized by intensive agricultural practices, demonstrate heightened OCP concentrations compared to Seixal and Alcochete salt marshes, influenced by industrial and urban sources [55,113]. The sediments of the Seixal salt marsh, situated in proximity to a former industrial area employing PCBs as coolants and lubricants, exhibit elevated PCB levels relative to the other two salt marshes [55]. Conversely, the sediments of the Alcochete salt marsh, positioned upstream of the other two marshes and within a protected wildlife reserve, manifest lower concentrations of both OCPs and PCBs, indicative of reduced contamination levels [114].

The findings of the present study confirm not only the continuous occurrence of OCPs in the salt marsh ecosystems of Alcochete, Rosário, and Seixal, but also reveal several key insights that can apply to other salt marsh ecosystems under similar anthropogenic pressure. The present OCP and PCB distribution patterns within our study therefore emphasize the need to recognize the historical and current human activities responsible for the pollutant levels at different sites. Having pinpointed these activities and their association with pollutant levels, such kinds of assessment can be done in other regions too, tailoring management strategies accordingly. In addition, the effect of site-specific sediment characteristics on pollutant concentrations indicates that these factors must also be taken into consideration in the design of management plans for other salt marsh ecosystems. Understanding how sediment properties affect pollutant levels will provide guidance for the choice and implementation of the right remediation methods and increase, ultimately, the effectiveness of the overall management effort. The findings in the current study, concerning *S. maritima* and OCP and PCB transport into this plant, are of general value, but also hold very significant implications for other salt marsh ecosystems. Phytostabilization processes of the pollutants in plants and metabolization provide help in understanding the basic concept of the selection of species in plant bioremediation and habitat restoration projects. Identification of plants that adequately reduce the pollutant concentration would improve the efficiency of conservation activities in the restoration and protection of ecosystems altered by human activities. Therefore, we highly encourage further research to better understand these processes, as this knowledge is key to the health and longevity of salt marsh systems globally and to develop more efficient management strategies.

The implementation of several management strategies can be considered: (1) source control: identifying and mitigating sources and transport of OCPs and PCBs, such as industrial discharge, urban runoff, and agricultural practices, and implementing best management practices and stricter regulations; (2) regular monitoring: establishing a long-term monitoring program to track contaminant levels in both sediments and plants; (3) habitat restoration: investing in restoration and conservation of salt marsh habitats [55] to enhance their natural resilience and ability to buffer against pollution; (4) public awareness and education: raising awareness about the impacts of PCBs and OCPs on the environment among local communities, industries, stakeholders and policymakers; and (5) collaborative management between researchers, environmental agencies, policymakers, and local stakeholders to develop and implement integrated coastal zone management plans, ensuring the long-term health of these ecosystems. Through the adoption of these strategies, we can work towards finding strategies to manage the levels of PCBs and OCPs in the Tagus estuary and other salt marsh ecosystems, ultimately safeguarding their biodiversity and ecosystem services for future generations.

## 5. Conclusions

This study has revealed the persistent presence of organochlorine compounds in the salt marsh ecosystems of Alcochete, Rosário, and Seixal, notwithstanding the regulatory prohibitions implemented in the 1990s, except for DDT, which was banned in the 1970s. Our findings indicate that Rosário exhibited the highest concentrations of OCPs, while Seixal displayed elevated levels of PCBs within its sediments. This distinctive distribution pattern corresponds to the historical anthropogenic activities characteristic of each locale. Rosário, historically associated with extensive agricultural practices, has a historical precedent for utilizing OCPs, whereas Seixal, as an industrial centre, employed PCB compounds. Conversely, sediment samples from Alcochete exhibited the lowest concentrations of these compounds. This observed variability can be directly attributed to sediment characteristics, particularly grain size. Comparisons of our results with analogous investigations conducted in diverse geographical locations underscored the pollution burden borne by the marshes investigated in this study, primarily attributable to their proximity to higher population densities. Comparing our sediment analysis with the SQG values established by the US EPA and OSPAR frameworks allowed us to establish a differential pattern across the estuary, highlighting the necessity for site-specific assessments and further investigations to pinpoint the sources and transport pathways contributing to these differences. An assessment of OCP and PCB concentrations within the *Spartina maritima* plant revealed the highest levels in belowground components, indicating that the transport of these compounds to aboveground segments occurs at lower concentrations. A minimal positive correlation between these two plant segments was observed. However, establishing a direct relationship for most of the analysed compounds in sediments and plant tissues (excluding DDE) proved challenging, implying a potential metabolization of the compounds within the plant tissues or the prospect of phytostabilisation of OCPs and PCBs in the sediments. This phenomenon contributes to the natural remediation of the system by mitigating the potential pathways for entry into the food chain. Based on these findings, the need for management strategies targeted at other salt marsh ecosystems facing similar pressures will require knowledge of historical and current human activities and specifics of sediment on-site characteristics. More intensive research on the sources and pathways of pollutants is needed to ensure the healthy and long-term conservation of such ecosystems. Furthermore, this study underlines how crucial it is to adopt a multidisciplinary approach to better preserve and restore salt marshes impacted by human activities.

## Figures and Tables

**Table 1:** Sediment quality guidelines (SQGs) values recommended by the US EPA and the OSPAR commission [67,68].

Compound	US EPA (µg Kg^−1^)	OSPAR (µg Kg^−1^)
PCB 28	–	1.7
PCB 52	–	2.7
PCB 101	–	3.0
PCB 118	–	0.6
PCB 138	–	7.9
PCB 153	–	40.0
PCB 180	–	12.0
ΣPCBs	11.50	–
ΣDDE	2.20	–
ΣDDT	1.58	–

## Data Availability

Data available upon request.

## Supplementary Materials

The following supporting information can be downloaded at: https://www.mdpi.com/article/10.3390/jox14030066/s1, Table S1: Conditions of the program used in the ASE technique; Table S2: Operating conditions of GC-ECD technique; Table S3: Peak retention time of each analyte (in minutes).
