# Supplementary material for: Persistent Organic Pollutants in Tagus Estuary Salt Marshes: Patterns of Contamination and Plant Uptake
Source: J Xenobiot. 2024 Sep 2;14(3):1165–86. doi: 10.3390/jox14030066 (PMC11417836; doi:10.3390/jox14030066)
Supplement: Supplementary file 1 [file jox-14-00066-s001.zip › jox-3070835-supplementary.pdf]

Supplemental Information

Table S1 – Conditions of the program used in the ASE technique.

Parameter	Experimental condition
Solvent	n-hexane:acetone (50:50, v/v)
Pressure	2000 psi
Temperature	100 ºC
Number of cycles	Two
Purge's time	180 s
Solvent volume	Half of the extraction cell's volume

Table S2 – Operating conditions of GC-ECD technique.

Component	Parameter	Condition
Syringe	Capacity	10 µL
Injector	Temperature	250 ºC
	Mode	Pulsed splitless
	Injected volume	2 µL
Furnace	Initial temperature	80 ºC (3 min)
	Heating ramp 1	30 ºC min^{-1} until 155 ºC
	Heating ramp 2	2 ºC until 188 ºC
	Heating ramp 3	3 ºC until 245 ºC
Carrier gas	Helium	20 mL min^{-1}
µECD detector	Temperature	320 ºC
	Electron capture	µECD-Ni^{63}
Detector's auxiliar gas	Argon/Methane	60 mL min^{-1}

Table S3 – Peak retention time of each analyte (in minutes).

Analyte	Retention time (min)
PCB 28	22.35
PCB 52	24.90
PCB 155	32.98
op' DDE	33.92
PCB 101	34.30
pp'- DDE	38.93
PCB 118	45.09
op' DDT	47.57
PCB 153	49.97
PCB 105	50.34
pp' DDT	53.87
PCB 138	54.35
PCB 156	60.82
PCB 180	62.69
PCB 198	66.00
